# Co-operation of MCL-1 and BCL-X_L_ anti-apoptotic proteins in stromal protection of MM cells from carfilzomib mediated cytotoxicity

**DOI:** 10.3389/fonc.2024.1394393

**Published:** 2024-04-08

**Authors:** Daria Galas-Filipowicz, Selina J. Chavda, Jia-Nan Gong, David C. S. Huang, Asim Khwaja, Kwee Yong

**Affiliations:** ^1^ Cancer Institute, University College London, London, United Kingdom; ^2^ Department of Medical Biology, University of Melbourne, Parkville, VIC, Australia; ^3^ Institute of Laboratory Animal Sciences, Chinese Academy of Medical Sciences & Peking Union Medical College, Beijing, China

**Keywords:** carfilzomib, drug resistance, stromal cells, anti-apoptotic proteins, Mcl-1, BCL-XL, BH3 mimetic

## Abstract

**Introduction:**

BCL-2 family proteins are important for tumour cell survival and drug resistance in multiple myeloma (MM). Although proteasome inhibitors are effective anti-myeloma drugs, some patients are resistant and almost all eventually relapse. We examined the function of BCL-2 family proteins in stromal-mediated resistance to carfilzomib-induced cytotoxicity in MM cells.

**Methods:**

Co-cultures employing HS5 stromal cells were used to model the interaction with stroma. MM cells were exposed to CFZ in a 1-hour pulse method. The expression of BCL-2 family proteins was assessed by flow cytometry and WB. Pro-survival proteins: MCL-1, BCL-2 and BCL-X_L_ were inhibited using S63845, ABT-199 and A-1331852 respectively. Changes in BIM binding partners were examined by immunoprecipitation and WB.

**Results:**

CFZ induced dose-dependent cell death of MM cells, primarily mediated by apoptosis. Culture of MM cells on HS-5 stromal cells resulted in reduced cytotoxicity to CFZ in a cell contact-dependent manner, upregulated expression of MCL-1 and increased dependency on BCL-X_L_. Inhibiting BCL-X_L_ or MCL-1 with BH-3 mimetics abrogated stromal-mediated protection only at high doses, which may not be achievable *in vivo*. However, combining BH-3 mimetics at sub-therapeutic doses, which alone were without effect, significantly enhanced CFZ-mediated cytotoxicity even in the presence of stroma. Furthermore, MCL-1 inhibition led to enhanced binding between BCL-X_L_ and BIM, while blocking BCL-X_L_ increased MCL-1/BIM complex formation, indicating the cooperative role of these proteins.

**Conclusion:**

Stromal interactions alter the dependence on BCL-2 family members, providing a rationale for dual inhibition to abrogate the protective effect of stroma and restore sensitivity to CFZ.

## Introduction

In multiple myeloma (MM), dependency on protein homeostatic mechanisms renders tumour cells vulnerable to proteasome inhibitors (PIs). Despite good initial responses to PIs, most MM patients eventually relapse, and some display primary resistance. The putative resistance mechanism remains incompletely understood, with several potential mechanisms suggested. Among them, the activation of the heat shock response stands out as one of the most prominent cytoprotective mechanisms (1). Mutations and abnormal expression of components within the ubiquitin-proteasome system are additional factors that can result in resistance to PIs ( (2–4). For instance, alterations in drug-binding sites might reduce the activity of PIs to inhibit proteolysis. Activation of the autophagy-lysosomal pathway to compensate for proteasome inhibition is another mechanism for resistance (5, 6). Upregulation of survivin, an apoptosis inhibitor, has been shown to play a role in decreased response to proteasome inhibition mainly by bortezomib (BTZ) (7). Finally, resistance to PIs may be because interactions with the bone marrow (BM) stromal environment promote resistance to apoptosis (8, 9).

The BCL-2 family of proteins regulates the intrinsic mitochondrial apoptotic pathway, comprising anti-apoptotic proteins (BCL-2, MCL-1, BCL-X_L_, BCL-W), pro-apoptotic BH-3 only proteins (BIM, NOXA, PUMA, BID), and pro-apoptotic effectors (BAX, BAK, BOK) (10). Within the BM, stromal cells secrete various cytokines (e.g. IL-6, IGF-1, VEGF) that can regulate the survival of plasma cells by altering dependence on anti-apoptotic members of the BCL-2 family. IL-6 modifies BIM through phosphorylation, increasing reliance on MCL-1 while reducing dependence on BCL-2/BCL-X_L_ (11).

Expression and function of BCL-2 family proteins in MM is therefore important for tumour cell survival and drug resistance and may partly mediate the protective effect of BM stroma. Recent clinical development of several inhibitors of anti-apoptotic proteins called BH-3 mimetic drugs has stimulated renewed interest in the function and regulation of these pro-survival proteins in MM.

Here, we investigated how anti-apoptotic proteins contribute to the protective function of stroma against CFZ-induced cytotoxicity and examined the interplay between different BCL-2 family members in the response of MM cells to carfilzomib.

## Methods

### Human myeloma cell lines (HMCL)

The following human myeloma cell lines, representing three most common IgH translocations in MM [t(14;16), t(4;14), t(11;14)] were used: MM1.s, RPMI8826, JJN3, H929, OPM2, JIM1, JIM3, KMS28, KMS12 BM, KMS27, U266, KMS21 BM (**Supplementary Table 1**). MM1.s Bax/Bak KO cells were generated using CRISPR/Cas9 (12). HMCLs were grown in suspension culture in RPMI 1640 medium (Lonza) with 10% Fetal Bovine Serum (FBS; Gibco) and 1% Penicillin/Streptomycin (Pen/Strep, Gibco).

### Stromal cells

HS5 stromal cells (ATCC) were grown as adherent monolayers in tissue culture flasks in Dulbecco’s Modified Eagle Medium (DMEM, Sigma) with 10% FBS/1% Pen/Strep, detached using trypsin/EDTA solution (Sigma) and subcultured. Cells were modified to express blue fluorescent protein (BFP) by lentiviral transduction (**Supplementary Figure 1A**).

### Co-culture of HMCL with HS5 BFP+ stromal cells

HS5 BPF+ were seeded into 48 well plates and grown to 80% confluence before the addition of HMCL (0.5x10^6^ cells/ml). To prevent disruption of the HS5 monolayer when harvesting MM cells, collagen-coated plates were used.

### Primary samples

Bone marrow aspirates were obtained from MM patients (49 newly diagnosed, 12 relapsed, 3 pre-ASCT), with written informed consent (REC 07/Q0502/17). BM mononuclear cells (MNCs) were isolated by Ficoll Paque (GE Healthcare) density centrifugation. MM cells were selected using CD138 MicroBeads (Miltenyi Biotec).

### Carfilzomib and BH-3 mimetics exposure

To recapitulate the pharmacokinetics of Carfilzomib (CFZ) in patients, MM cells were exposed to CFZ (10-400nM) for 1 hour and then washed twice with RPMI, resuspended in drug-free medium, and plated alone or co-cultured with HS5 BFP+ cells.

The following BH-3 mimetics were used: MCL-1 inhibitors: S63845 (CymanChem), AZD5991 (Stratech Scientific), BCL-X_L_ inhibitor: A-1331852 (Abcam), BCL-2 inhibitor: ABT-199 (Venetoclax; BioVision), BCL-2/BCL-X_L_ dual inhibitor: ABT-263 (Navitoclax; Abcam).

### Apoptosis assay

Cell death was assessed by Annexin V APC (BD Bioscience)/Propidium Iodide (Sigma) staining and flow cytometry. Cells were co-stained with AnV/PI for 15 minutes at 4°C, washed in 1ml PBS/0.5%BSA buffer and resuspended in 0.4ml PBS/0.5%BSA containing Flow Check Beads (Beckman Coulter). The percentage of cell viability was calculated by normalising the values against their corresponding untreated controls.

### Surface antigen and intracellular staining

CD138 immunostaining was used to identify MM plasma cells in patient MNCs. MNCs were washed in PBS/0.5%BSA buffer, stained with 1µl of CD138 APC (BioLegend) antibody for 30 mins at 4°C, washed and resuspended in 400µl of PBS/0.5% BSA buffer and 10µl Flow Check Beads. Live cell numbers were quantified by reference to the Flow Check Beads. Intracellular staining for BCL-2 family proteins was performed using the Fixation/Permeabilization Solution Kit (BD Biosciences).

### Western blot analysis

Total protein cell lysates were prepared using RIPA lysis buffer (Merck Millipore) containing proteinase inhibitors on ice. 30µg of protein was loaded per condition and separated on a 4-12% NuPage Gel (Invitrogen), transferred to a PVDF membrane (semi-dry transfer), blocked with 5% skimmed milk in TBS-Tween, and probed overnight with primary antibodies, washed, and exposed to HRP-conjugated secondary antibody for 1 hour. Chemiluminescence was developed using the Lumianta Forte HRP (Merck Millipore) substrate. Images were taken using BioRad ChemiDoc imaging system and analysed by ImageJ software.

### RNA extraction and gene expression analysis

RNA was extracted using RaliaPrep™ RNA Cell Miniprep System (Promega). cDNA was prepared by reverse transcription of purified RNA using SuperScript™ IV RT kit (Invitrogen). qPCR was performed with QuantStudio5 (applied biosystems by Thermo Fisher Scientific) using gene-specific primers (sequences listed in **Supplementary Methods**) and SYBBR Green Master Mix (Roche). Delta- delta Ct method was used to analyse the qPCR data.

### Immunoprecipitation (IP)

Cells were washed in ice-cold PBS and lysed in 1x Pierce™ IP Lysis Buffer (ThermoScientific) supplemented with a protease/phosphatase inhibitor cocktail (Roche). 500µg of total protein was incubated with BIM IP Ab (Cell Signaling) overnight (4°C), then with 25µl of protein G agarose beads (Cell Signaling) for 1 hour. Beads were washed with ice-cold 1x IP Lysis Buffer and boiled with 25µl of LDS sample buffer for 10 minutes. Samples were then separated by SDS-PAGE and analysed by western blotting.

### Data analysis

FlowJo software was used for flow cytometry data analysis. GraphPad Prism 9 was used for the graphical presentation of data and statistical analysis. All data are expressed as means ± SEM and analysed by analysis of variance (ANOVA) or Student t test for statistical significance. P values <0.05 were deemed statistically significant.

## Results

### MM cells display variable sensitivity to CFZ killing and uniform protection by stroma

Firstly, we investigated CFZ-induced cytotoxicity, and the effect of stroma co-culture in HMCLs representing the three most common recurrent translocations involving the IgH locus (t(4;14), t(14; 16), t(11;14)).

**Figure 1:**
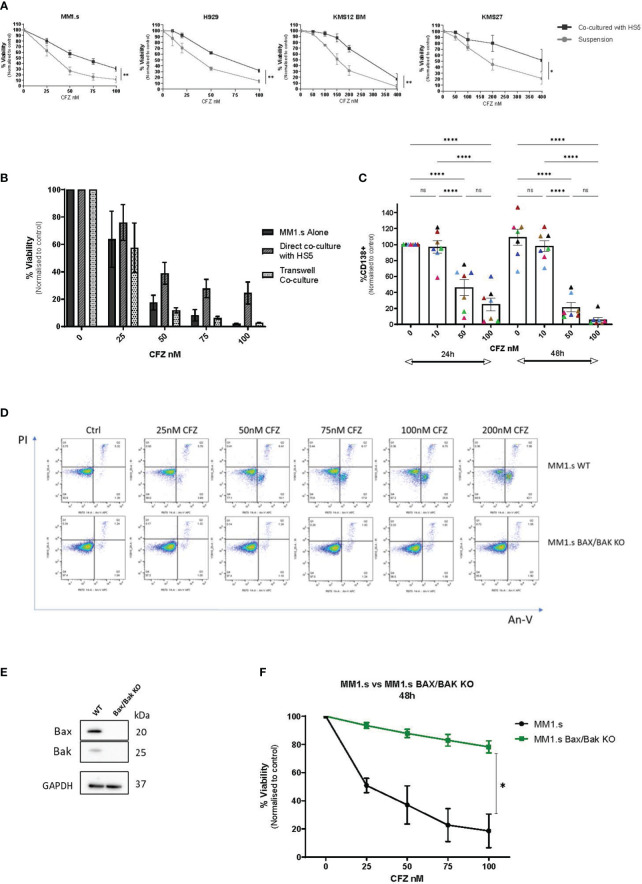
CFZ cytotoxicity in MM cells and effect of stromal co-culture. **(A)** MM cell lines were cultured alone or co-cultured with HS5 monolayers for up to 48 hours following CFZ treatment. Cell viability was assessed by Annexin V/PI staining. Data are presented as mean +/- SEM of 3 independent experiments. **(B)** MM1.s cells were treated with 1h pulse CFZ, washed and cultured alone, on stroma (direct co-culture) or transwells for 48h and viability was assessed by AnV/PI staining. Data are presented as mean +/- SEM of 3 independent experiments. **(C)** Primary samples (MNCs) derived from myeloma patients (N=7) were treated with CFZ and left in culture for 24h and 48h. The loss of CD138+ MM cells was assessed by flow cytometry following staining with CD138 Ab. 2-way ANOVA with Tukey’s multiple comparisons test. **(D)** MM1.s WT and MM1.s Bax/Bak KO cells were treated with indicated doses of CFZ. Cell viability was assessed at 6h using AnV/PI staining. Representative flow cytometry plots are shown. **(E)** Validation of CRISPR/Cas9 KO of Bax and Bak in MM1.s by Western Blot. **(F)** MM1.s WT and MM1.s Bax/Bak KO cells were treated with increasing doses of CFZ (0-100nM) and cell death was assessed after 48h. 2-tailed t-test; p=0.04. Data are presented as mean +/- SEM of 3 independent experiments. * - p<0.05; **- p<0.005; ****- p<0.0005; ns - not significant.

CFZ induced dose-dependent cell death in all HMCLs tested (**Figure 1A**). MM1.s [t(14;16)] cells showed the greatest sensitivity to CFZ (IC50 = 25.2nM), compared to other HMCL, where higher CFZ concentrations were needed to induce similar levels of cell death. In all four cell lines, cell death was significantly reduced when co-cultured with HS5 stromal cells (**Figure 1A**). When MM cells were separated from HS5 cells by transwells, this protection was not seen, indicating a requirement for direct “cell-to-cell” contact (**Figure 1B**).

Next, we examined primary bone marrow (BM) CD138+ cells for their susceptibility to carfilzomib-induced apoptosis (**Figure 1C**, **Supplementary Table 3**). Cultures were set up as whole BM MNCs, with the MM cells in proximity to the non-tumour compartment, including stromal cells. Although the early (24h) response was variable between patients, all samples tested showed a dose and time-dependent loss of CD138+ cells with a reduction to 22 ± 6% of control (n=7 p<0.0001) at 48h post exposure to 50nM of CFZ. We did not observe a significant difference between samples derived from patients representing adverse [(t(4;14), n=4] and standard risk [t(11;14), n=3] cytogenetics. Notably, the best and worst responders to CFZ, both had t(4;14) translocation (**Figure 1C**, pink and black triangles, respectively), but their disease status differed: the first was newly diagnosed untreated MM, and the second was fourth relapsed MM with multiple prior treatments.

### CFZ-mediated killing occurs via the intrinsic apoptotic pathway

To further investigate the role of stroma in CFZ-mediated cytotoxicity, we used MM1.s as a model HMCL. Apoptosis was assessed by detection of fluorescently labelled Annexin V, which binds to phosphatidylserine when it is on the outer leaflet of the plasma membrane. CFZ cytotoxicity was primarily mediated by apoptosis in MM1.s WT as shown by AnV/PI staining (**Figure 1D**, top panel), with a dose-dependent shift from AnV^-^/PI^-^ population (live cells) towards AnV^+^/PI^-^ (early apoptotic cells) and subsequently AnV^+^/PI^+^ (late apoptotic cells) populations. Additionally, we used MM1.s BAX/BAK KO cells (**Figure 1E**), to investigate CFZ-mediated killing in the context of genetic deletion of apoptosis effectors. MM1.s Bax/Bak KO cells did not show the same apoptotic pattern (**Figure 1D**, bottom panel) as WT cells. We observed significantly reduced cytotoxicity in MM1.s BAX/BAK KO cells, compared to MM1.s WT (**Figures 1D, F**). 25nM CFZ resulted in 7 ± 2% cell death in KO cells compared to 49 ± 5% in MM1.s WT at 48h (p<0.0001, n=3), with even more marked difference observed at higher doses of CFZ (100nM: 22 ± 4% vs 82 ± 11% at 48h, p<0.0001, n=3). These results indicate that killing of MM1.s cells by CFZ occurs largely via mitochondrial-mediated apoptosis.

### Expression and function of BCL2 family proteins in MM cells

**Figure 2:**
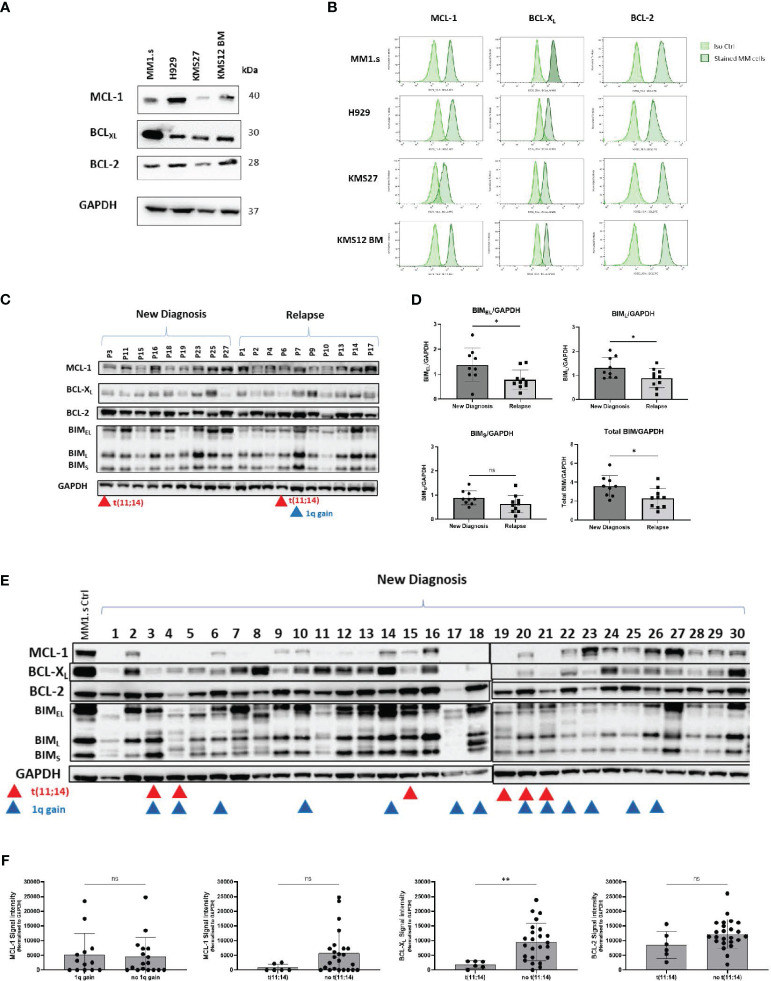
Expression of anti-apoptotic proteins in MM cell lines and primary samples. **(A)** Total protein of MM cell lysates was extracted and subjected to Western Blot analysis. GAPDH was used as an internal control. **(B)** MM cell lines were subjected to intracellular staining with BCL-X_L_, BCL-2 and MCL-1 antibodies and expression of anti-apoptotic proteins was evaluated by flow cytometry. Representative flow plots are shown. **(C)** Total protein lysates of primary CD138+ cells (New Diagnosis: n=9, Relapse: n=10) were subjected to Western Blot analysis of anti-apoptotic proteins: MCL-1, BCL-X_L_, BCL-2, and pro-apoptotic protein BIM. Samples from patients with t(11;14), and 1q gain are marked by red and blue triangles respectively. **(D)** Comparison of densitometry quantification of BIM isoforms between newly diagnosed and relapsed patients. Data are presented as BIM/GAPDH ratio. Analysis was performed using ImageJ software. **(E)** Total protein lysates of primary CD138+ cells (New Diagnosis: n=30), were subjected to Western Blot analysis of anti-apoptotic proteins: MCL-1, BCL-X_L_, BCL-2, and pro-apoptotic protein BIM. Samples from patients with t(11;14), and 1q gain are marked by red and blue triangles respectively. **(F)** Comparison of densitometry quantification of MCL-1 expression between patients with 1q and no 1q gain; and MCL-1, BCL-X_L_ and BCL-2 expression for patients with t(11;14) vs not(11;14). Statistics performed using unpaired t-test **p=0.0097.

We next evaluated the expression of pro-survival BCL-2 family proteins in a panel of MM cell lines by Western blot (**Figure 2A**) and by flow cytometry (**Figure 2B**). The expression of pro-survival proteins was heterogeneous with the highest BCL-X_L_ level in MM1.s cells (WB and Flow) and prominent BCL-2 expression in KMS12 BM, consistent with previous reports on MM cells with t(11;14) (4, 13).

Expression of anti-and pro-apoptotic proteins was also heterogeneous across primary MM cells (**Figures 2C, E**, **Supplementary Table 4**). We detected significantly lower levels of pro-apoptotic BIM_EL_ and BIM_L_ proteins in samples from relapsed patients, compared to samples from newly diagnosed patients (p=0.028), perhaps contributing to treatment resistance (**Figure 2D**). We further investigated the expression of anti-apoptotic proteins in thirty newly diagnosed samples (**Figure 2E**). BCL-2 was readily detected in nearly all cases and levels did not correlate with the presence of t(11;14). BCL-X_L_ and MCL-1 expression was variable and was low/absent in t(11;14) samples, consistent with a predominant reliance on BCL-2, and venetoclax sensitivity in this group of patients (**Figure 2F**) (14). The *MCL-1* gene is located on 1q21, but we did not find any correlation between 1q gain and expression of MCL-1 in our cohort (**Figure 2F**).

**Figure 3:**
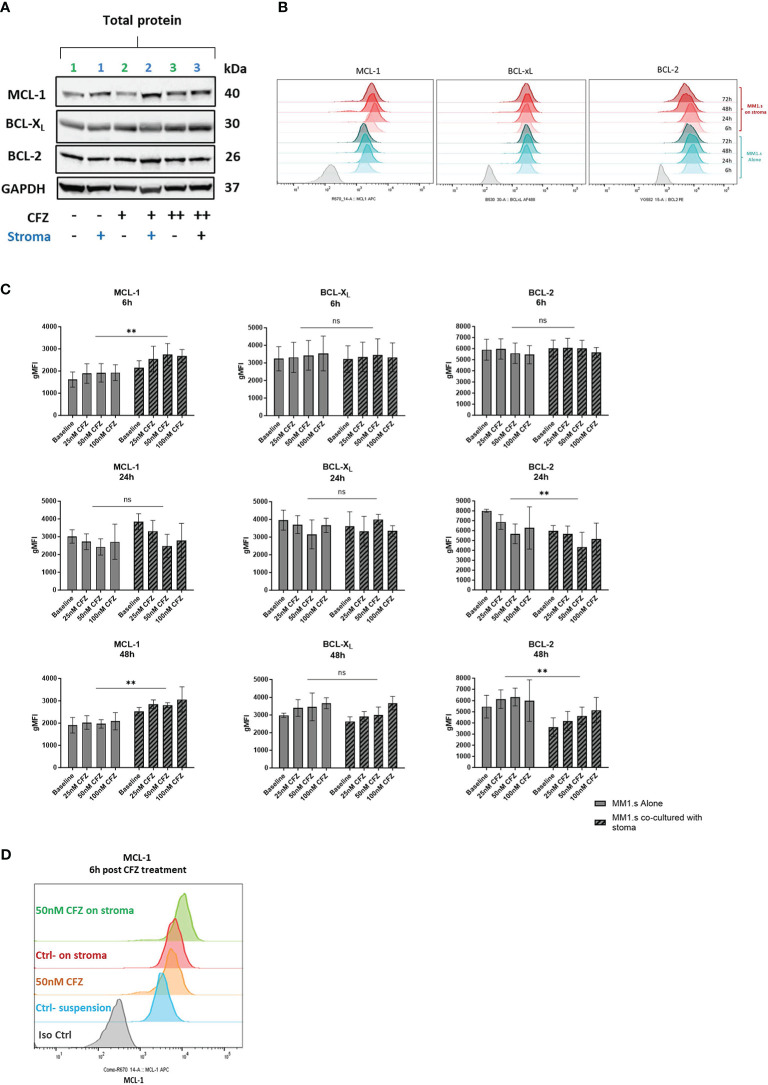
Expression of pro-survival proteins after CFZ exposure and effect of stroma. **(A)** MM1.s cells were treated with 25nM (line 2) and 100nM (line 3) CFZ for 1h, washed and left in suspension or co-cultured with HS5 for 6h. Total protein lysates from MM1.s cells were subjected to Western Blot analysis. Representative western blot reflecting changes in protein expression is shown. **(B)** MM1.s cells were co-cultured alone and on stroma for up to 72h. Changes in the expression of pro-survival proteins post-stroma co-culture were analysed by flow cytometry. Representative histograms are shown. **(C)** MM1.s cells were co-cultured alone and on stroma for 6h and 48h. Changes in the expression of pro-survival proteins were analysed by flow cytometry. gMFI = geometric Mean Fluorescence Intensity. Data are presented as mean +/- SEM of 3 independent experiments. **(D)** MM1.s cells were treated with 50nM CFZ for 1h, washed, cultured alone or on stroma for 6h and analysed by flow cytometry. Representative histograms are shown. ** p<0.005; ns, not significant.

Next, we examined the relative contribution of the different anti-apoptotic proteins to MM cell survival. We observed that most HMCLs are mainly dependent on MCL-1 for survival, as indicated by dose-dependent cytotoxicity of the MCL-1 specific BH3 mimetic AZD5991 in suspension cultures (**Supplementary Figure 2A**). The majority of HMCLs were resistant to the BCL-2 inhibitor, ABT-199 (**Supplementary Figure 2B**), despite expression of BCL-2 protein, e.g., MM1.s and H929 (**Figure 3A**). Interestingly, KMS27 and KMS12 BM, both harbouring t(11;14), were among three HMCLs to show sensitivity to ABT-199, indicating BCL-2 dependence. MM1.s cells being particularly resistant to MCL-1 and BCL-2 inhibition, were sensitive to ABT-263 (navitoclax) (**Supplementary Figure 3**), a dual inhibitor of BCL-2 and BCL-X_L_, suggesting BCL-X_L_ dependence for this cell line. This was supported by use of the BCL-X_L_ specific inhibitor A-1331852, which induced dose-dependent cell death in MM1.s (**Supplementary Figure 3**).

### Exposure of MM cells to CFZ in stromal co-cultures leads to upregulation of MCL-1 and increased dependency on BCL-X_L_

We then investigated changes in expression of anti-apoptotic proteins in response to stroma and CFZ treatment. We performed initial analysis by Western blot (**Figure 3A**) and used flow cytometry to quantify MCL-1, BCL-X_L_ and BCL-2 expression. We observed a rapid increase in MCL-1 levels post CFZ exposure (50 and 100nM), which is consistent with the fact that MCL-1 has a short half-life (~30min), being regulated by proteasomal degradation (**Figures 3A, B, D**). Expression of BCL-X_L_ and BCL-2 were also increased at 48h in suspension cultures (**Figure 3C**).

Co-culture of MM1.s with HS-5 cells induced upregulation of MCL-1 and downregulation of BCL-2 (**Figure 3C**). Additionally, exposure of MM cells to CFZ in stromal co-cultures led to a further increase in the expression of MCL-1 (**Figures 3C, D**). These changes were observed as early as 6h post co-culture with stroma. Examining transcripts, we found no significant change in *MCL-1* mRNA expression, while both *BCL-XL* and *BCL-2* were downregulated (**Supplementary Figure 4**). The data indicates that the increase in MCL-1 protein seen on stroma is not mediated via altered transcription, unlike BCL-2, which exhibited notably reduced levels at 6- and 24-hours following stroma co-culture.

### Effect of BH3 mimetics on stromal protection against CFZ-mediated cytotoxicity

We used selective inhibitors of MCL-1 (S63845), BCL-X_L_ (A-1331852) and BCL-2 (ABT-199) to investigate the role of these anti-apoptotic proteins in mediating the protective effect of stroma. In MM1.s cells, inhibiting either MCL-1 or BCL-X_L_ in absence of stroma enhanced CFZ-induced cytotoxicity in a dose-dependent manner, an effect that was significantly reduced by stroma (**Supplementary Figure 5A**). This protective effect of stroma could be abrogated by A-1331852, but only at high doses, which may not be achievable *in vivo* (**Supplementary Figure 5C**), due to human platelets’ dependence on BCL-X_L_ for survival (15). At lower doses, however, the effect of a single inhibitor on CFZ-induced cytotoxicity was minimal and did not alter the proportionate protection by stroma (**Supplementary Figures 5D, E**).

**Figure 4:**
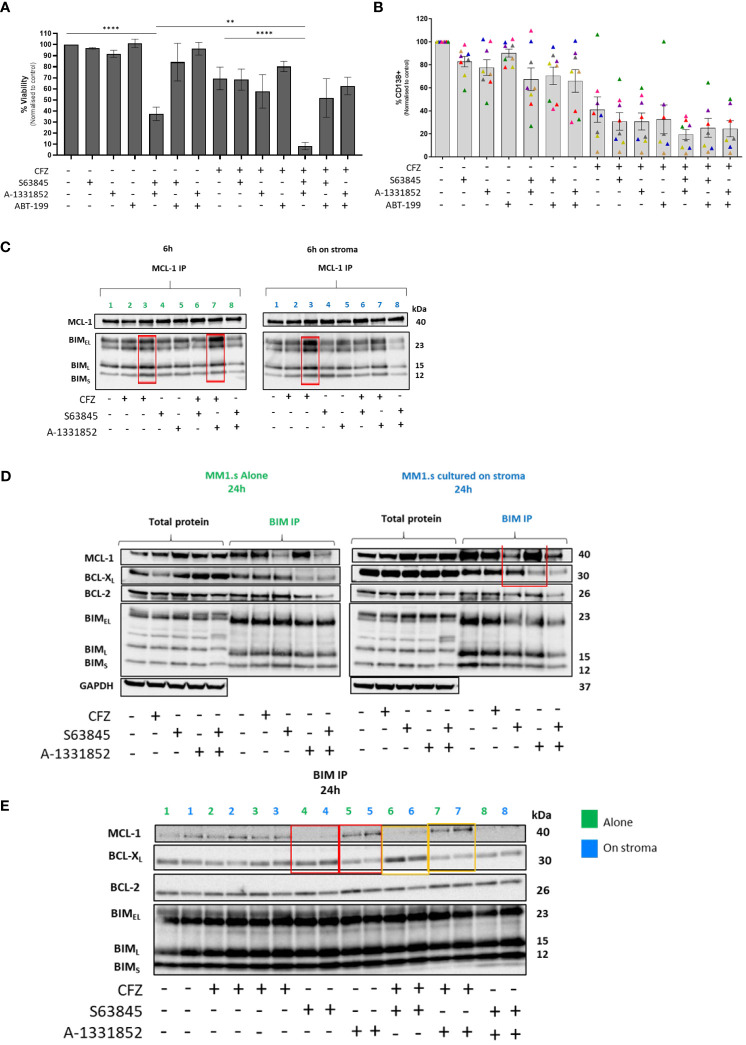
Effect of combining BH-3 mimetics with CFZ in MM cells co-cultured with HS5 and cooperativity of anti-apoptotic proteins on resistance to CFZ. **(A)** 48h co-cultures of MM1.s with HS5 were set up, following treatment with 25nM CFZ. BH-3 mimetics (100nM S63845, 1µM ABT-199, 1nM A-1331852) were added to co-cultures. Data are presented as mean +/- SEM of 3 independent experiments. p<0.05 deemed statistically significant.**** p<0.0001, **p=0.01 **(B)** The combination of CFZ (25nM) and BH-3 mimetics (10nM) tested on MNCs derived from newly diagnosed MM patients (n=8). The loss of CD138+ MM cells was measured by flow cytometry following staining with CD138 Ab. Note: Patient marked with a pink triangle is missing three data points (not enough cells). **(C)** MCL-1 IP- Protein lysates from MM1.s were subjected to MCL-1 immunoprecipitation. Protein lysates were prepared 6h post-CFZ and BH-3 mimetics exposure **(D, E)** BIM IP- Protein lysates from MM1.s were subjected to BIM immunoprecipitation. Protein lysates were prepared 24h post-CFZ and BH-3 mimetics exposure. Line: 1.Ctrl; 2. 25nM CFZ; 3.100nM CFZ; 4.100nM S63845 (MCL-1 inhibitor); 5.1nM A-1331852 (BCL-X_L_ inhibitor); 6.25nM CFZ+ S63845; 7.25nM CFZ+ A-1331852; 8. S63845+ A-1331852.

We therefore reasoned that increased MCL-1 expression in stromal co-culture may result in a greater dependence on this protein, but only when other anti-apoptotic pathways are blocked. When used in combination at suboptimal concentrations (100nM S63845 and 1nM A-1331852), that did not substantially affect cell viability as single agents, these BH3 mimetics potently sensitized MM cells to CFZ-mediated cytotoxicity in the presence of stroma, and indeed resulted in substantially greater cell death even in absence of CFZ (**Figure 4A**, **Supplementary Figure 6A**). In contrast, BCL-2 inhibition was without effect, either alone or in combination with either MCL-1 or BCL-X_L_ inhibitors. When tested on primary CD138+ plasma cells from newly diagnosed MM patients (**Table S5**), we also observed that combination of two BH3 mimetics and CFZ was the most effective in reducing viable cell number of malignant plasma cells (**Figure 4B**). As an example, for patient one (**Supplementary Figure 6B**), dual inhibition of MCL-1 and BCL-X_L_ was the only combination that significantly increased cell death and enhanced the CFZ response. These data indicate that combination of BH3 mimetics at subtherapeutic doses with PIs may be a potential therapeutic strategy, but the choice of effective inhibitor combination may vary between patients. Limited data suggest that dual MCL-1/BCL-X_L_ inhibition would be broadly effective, but larger patient cohorts are required to validate the superior effectiveness.

### Switch in binding activity of BIM between MCL-1 and BCL-X_L_ upon cellular stress

Because of the potent effect of dual MCL-1/BCL-X_L_ inhibition on killing MM cells even in the presence of stroma, we examined the binding of these anti-apoptotic proteins to the pro-apoptotic protein, BIM, in cells exposed to CFZ in the presence of stroma. MM1.s cells were treated with CFZ and protein lysates made at 6 hours post CFZ exposure and subjected to MCL-1 immunoprecipitation to evaluate the relative BIM binding to this anti-apoptotic protein. We observed that CFZ increased BIM binding to MCL-1 in a dose-dependent manner, which was further augmented by BCL-X_L_ inhibition (lanes 3 and 7, **Figure 4C**).

We also performed BIM immunoprecipitation to investigate its binding partners under different treatment conditions. This showed that in the presence of the MCL-1 inhibitor, S63845, there was increased BCL-X_L_ binding to BIM; conversely when BCL-X_L_ was inhibited by A-1331852, an increase in MCL-1/BIM complexes was seen (highlighted in red, **Figures 4D, E**). Interestingly, BCL-2 binding remained unchanged, suggesting that it is not taking over the role if both MCL-1 and BCL-X_L_ are inhibited. The same effect was seen for samples treated with CFZ and S63845/A-1331852 combination, with or without stromal co-culture (lanes 6-7, highlighted in yellow, **Figure 4E**). These results indicate the switch in survival dependency between MCL-1 and BCL-X_L_, which could be abolished by dual inhibition of these pro-survival proteins (line 8, **Figure 4E**).

## Discussion

In this study, the role of BCL-2 family members in stroma-mediated resistance from carfilzomib killing was investigated. We have confirmed the heterogenous expression of BCL-2 family proteins and the importance of MCL-1 and BCL-X_L_ in MM cell survival. Combined BCL-X_L_ and MCL-1 inhibition, using sub-therapeutic concentrations of BH3 mimetics, potently sensitized MM cells to CFZ-mediated cytotoxicity, abrogating stromal protection. Our findings suggest that this is related to a switch in binding activity of BIM between BCL-X_L_ and MCL-1 in the presence of BH3 mimetics.

As CFZ-induced cytotoxicity was primarily mediated by apoptosis, we focussed on the expression and function of BCL-2 family proteins. Overexpression of pro-survival proteins has been observed in MM, but Bcl-2 family dependence is highly variable (16), as we confirm here. Although samples with t(11;14) expressed high levels of BCL-2 with relatively lower expression of MCL-1 and BCL-X_L_, expression of BCL-2 was seen across all tested samples, not being restricted to those with t(11;14), as previously reported (13, 17).

We used cell contact co-cultures with the human stromal cell line, HS5, to investigate the function of anti-apoptotic proteins in mediating the relative resistance to CFZ when MM cells are in contact with stroma. We demonstrate upregulation of MCL-1 and increased dependency on BCL-X_L_ in presence of stroma, consistent with previous reports (10).

Stromal cells in the bone marrow secrete cytokines (IL-6, BAFF, APRIL, IFN-a) that promote plasma cell survival by regulating anti-apoptotic members of the BCL-2 family including MCL-1, BCL-X_L_ and BCL-2 (18). Upregulation of MCL-1 could result from the ligation of cell surface adhesion molecules and subsequent release of cytokines in our cell: cell contact system (19).

While inhibiting either BCL-X_L_ or MCL-1 alone had limited impact on CFZ-induced cytotoxicity, combined inhibition using sub-therapeutic doses significantly enhanced CFZ-mediated cytotoxicity, abrogating stromal protection. Our findings are consistent with results from Bolomsky et al., where the efficacy of MCL-1 inhibition in stromal settings was enhanced by concurrent BCL-X_L_ or BCL-2 inhibition (19). Ponder et al. have shown the importance of MCL-1 in MM cell survival and proposed combining carfilzomib (which increases Noxa mRNA expression) with TG02 (decreases MCL-1 protein) as a strategy for dual MCL-1 inhibition (20). This combination was also effective in stromal co-culture settings.

MCL-1 inhibition using S63845 increased BCL-X_L_ binding to BIM; conversely, the BCL-X_L_ inhibitor, A-1331852 resulted in increased MCL-1/BIM complexes. This ability to switch BIM binding was also seen in suspension cultures, however, the dependency on MCL-1 for BIM binding appears to be enhanced on stroma. Of note, the binding of BCL-2 to BIM stayed unchanged in all conditions, indicating that it may not assume an anti-apoptotic role if both MCL-1 and BCL-X_L_ are inhibited.

BH3 mimetics as a monotherapy may be ineffective as MM cells can alter their reliance on specific anti-apoptotic proteins, hence dual inhibition may be more effective (21). The AZ5991/venetoclax combination has been shown to overcome MCL-1 resistance (22) and dexamethasone aids MM cytotoxicity by promoting BCL-2 dependence via increased BCL-2 and BIM expression (23). Simultaneous inhibition of BCL-2 and MCL-1 using venetoclax and S63845 increased apoptotic cell death and reduced cell viability (24).

Combinations of BH-3 mimetic drugs may increase the efficacy of proteasome inhibitors in MM. Our results provide a rationale for exploring MCL-1 and BCL-X_L_ inhibition in MM, especially when used at lower, better-tolerated doses and in combination with proteasome inhibitors. Cooperativity between different Bcl-2 family members may differ according to genetic lesion and disease subtype, as seen in both HMCL and primary MM cells. Larger patient cohorts are required to confirm these findings and to characterise features responsible for sensitivity to a particular inhibitor.

## Data availability statement

The raw data supporting the conclusions of this article will be made available by the authors, without undue reservation.

## Ethics statement

The studies involving humans were approved by NHS Health Research Authority London-Harrow Research Ethics Committee REC 07/Q0502/17. The studies were conducted in accordance with the local legislation and institutional requirements. The participants provided their written informed consent to participate in this study.

## Author contributions

DG-F: Conceptualization, Data curation, Investigation, Methodology, Writing – original draft, Writing – review & editing. SC: Writing – review & editing, Methodology. J-NG: Methodology, Resources, Writing – review & editing. DH: Methodology, Resources, Writing – review & editing. AK: Conceptualization, Supervision, Writing – review & editing. KY: Conceptualization, Funding acquisition, Supervision, Writing – original draft, Writing – review & editing.
