# Supplementary material for: Co-operation of MCL-1 and BCL-XL anti-apoptotic proteins in stromal protection of MM cells from carfilzomib mediated cytotoxicity
Source: Front Oncol. 2024 Apr 8;14:1394393. doi: 10.3389/fonc.2024.1394393 (PMC11033393; doi:10.3389/fonc.2024.1394393)
Supplement: Supplementary file 1 [file Presentation_1.pptx]

## Slide 1
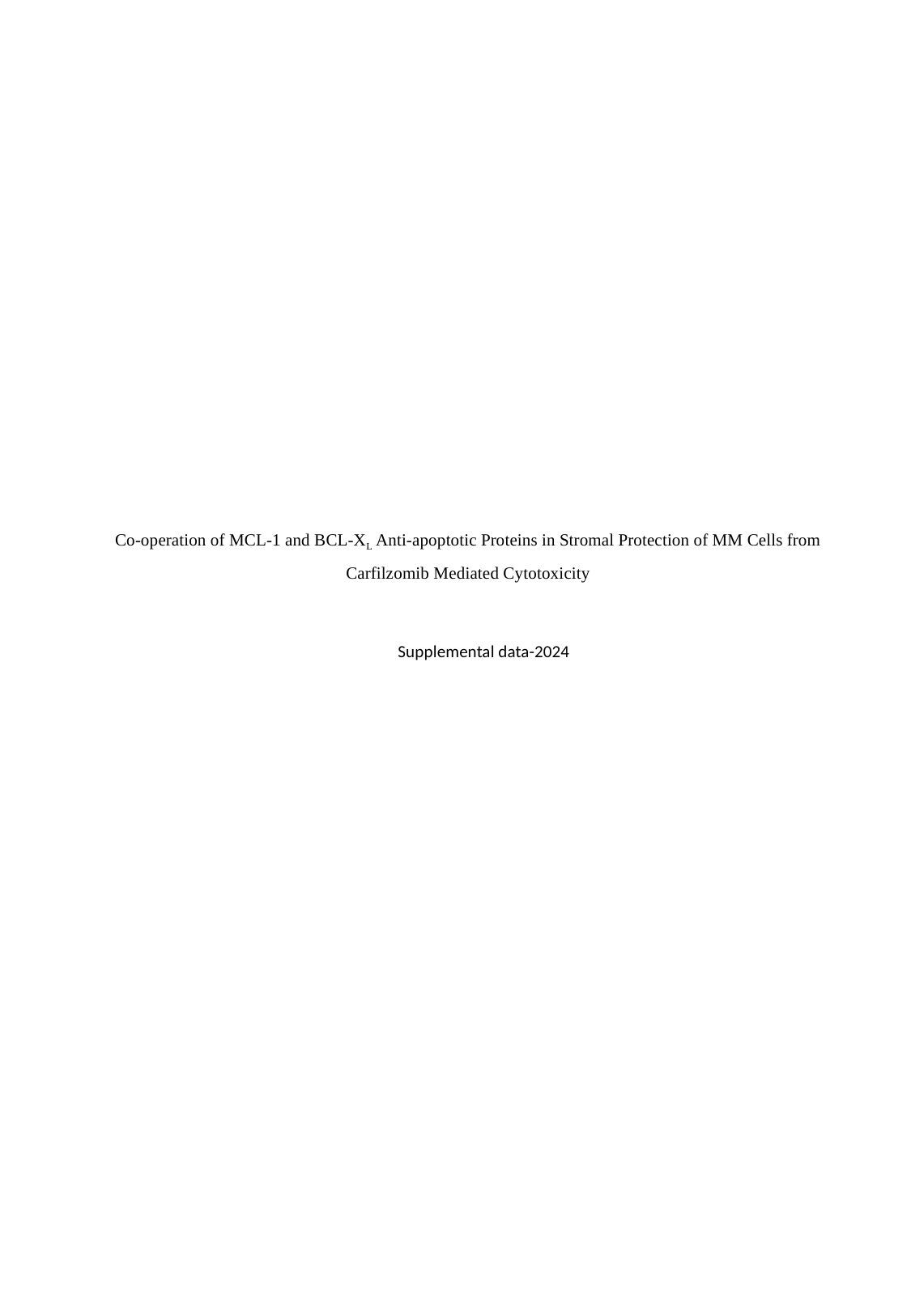

Co-operation of MCL-1 and BCL-XL Anti-apoptotic Proteins in Stromal Protection of MM Cells from Carfilzomib Mediated Cytotoxicity
Supplemental data-2024

## Slide 2
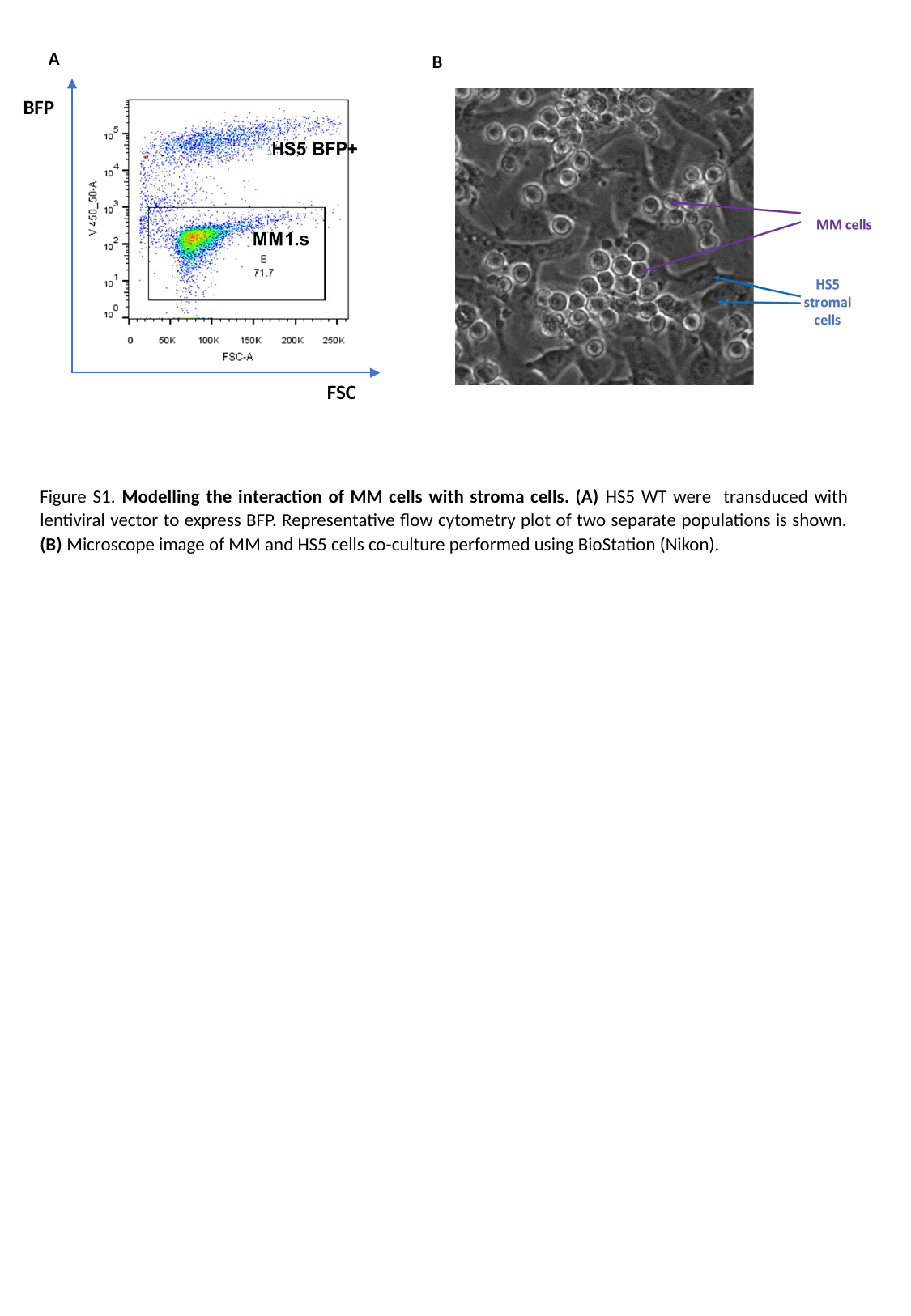

A
B
BFP
FSC
Figure S1. Modelling the interaction of MM cells with stroma cells. (A) HS5 WT were transduced with lentiviral vector to express BFP. Representative flow cytometry plot of two separate populations is shown. (B) Microscope image of MM and HS5 cells co-culture performed using BioStation (Nikon).

## Slide 3
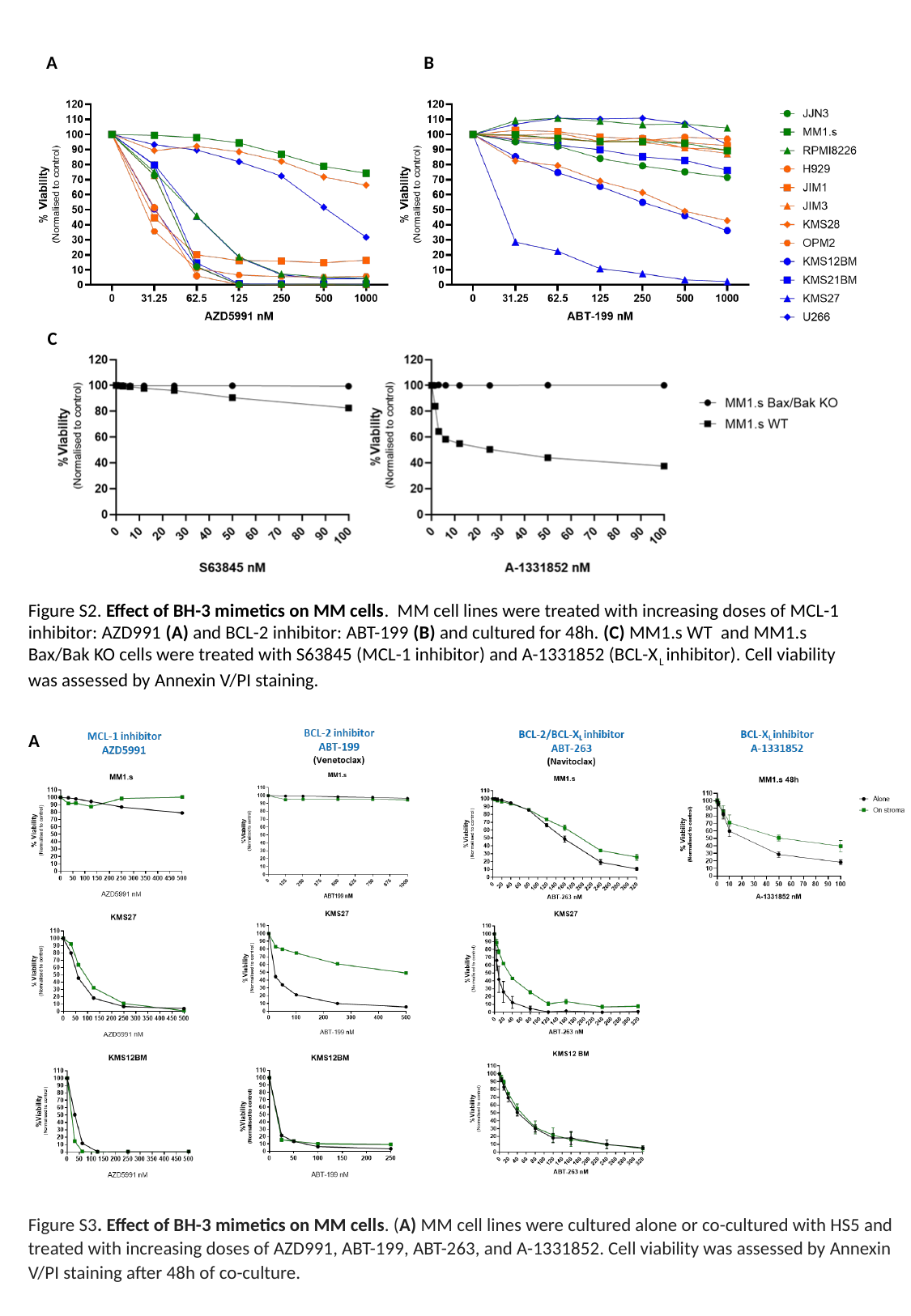

A
B
C
Figure S2. Effect of BH-3 mimetics on MM cells. MM cell lines were treated with increasing doses of MCL-1 inhibitor: AZD991 (A) and BCL-2 inhibitor: ABT-199 (B) and cultured for 48h. (C) MM1.s WT and MM1.s Bax/Bak KO cells were treated with S63845 (MCL-1 inhibitor) and A-1331852 (BCL-XL inhibitor). Cell viability was assessed by Annexin V/PI staining.
A
Figure S3. Effect of BH-3 mimetics on MM cells. (A) MM cell lines were cultured alone or co-cultured with HS5 and treated with increasing doses of AZD991, ABT-199, ABT-263, and A-1331852. Cell viability was assessed by Annexin V/PI staining after 48h of co-culture.

## Slide 4
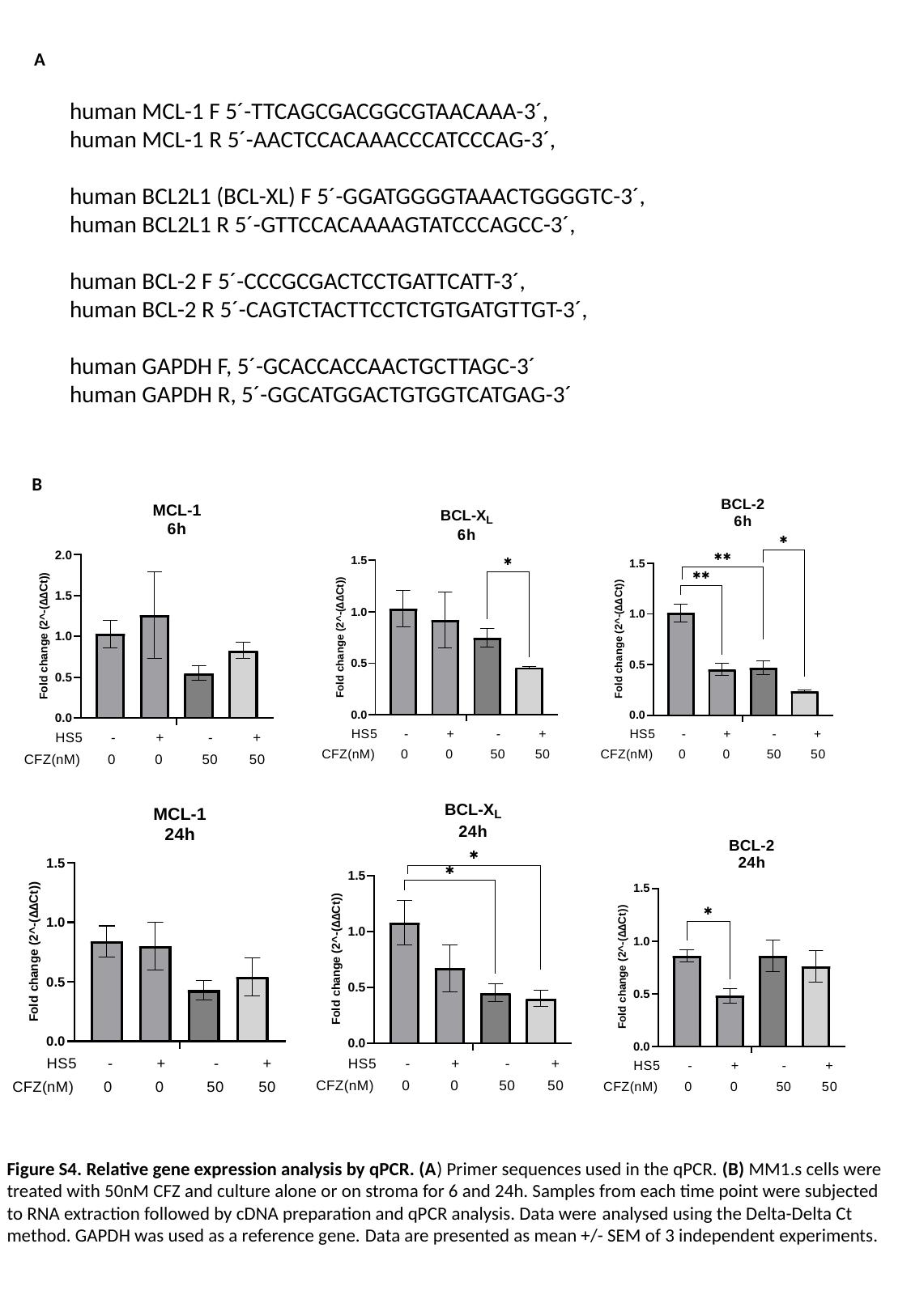

A
human MCL-1 F 5´-TTCAGCGACGGCGTAACAAA-3´,
human MCL-1 R 5´-AACTCCACAAACCCATCCCAG-3´,
human BCL2L1 (BCL-XL) F 5´-GGATGGGGTAAACTGGGGTC-3´,
human BCL2L1 R 5´-GTTCCACAAAAGTATCCCAGCC-3´,
human BCL-2 F 5´-CCCGCGACTCCTGATTCATT-3´,
human BCL-2 R 5´-CAGTCTACTTCCTCTGTGATGTTGT-3´,
human GAPDH F, 5´-GCACCACCAACTGCTTAGC-3´
human GAPDH R, 5´-GGCATGGACTGTGGTCATGAG-3´
B
Figure S4. Relative gene expression analysis by qPCR. (A) Primer sequences used in the qPCR. (B) MM1.s cells were treated with 50nM CFZ and culture alone or on stroma for 6 and 24h. Samples from each time point were subjected to RNA extraction followed by cDNA preparation and qPCR analysis. Data were analysed using the Delta-Delta Ct method. GAPDH was used as a reference gene. Data are presented as mean +/- SEM of 3 independent experiments.

## Slide 5
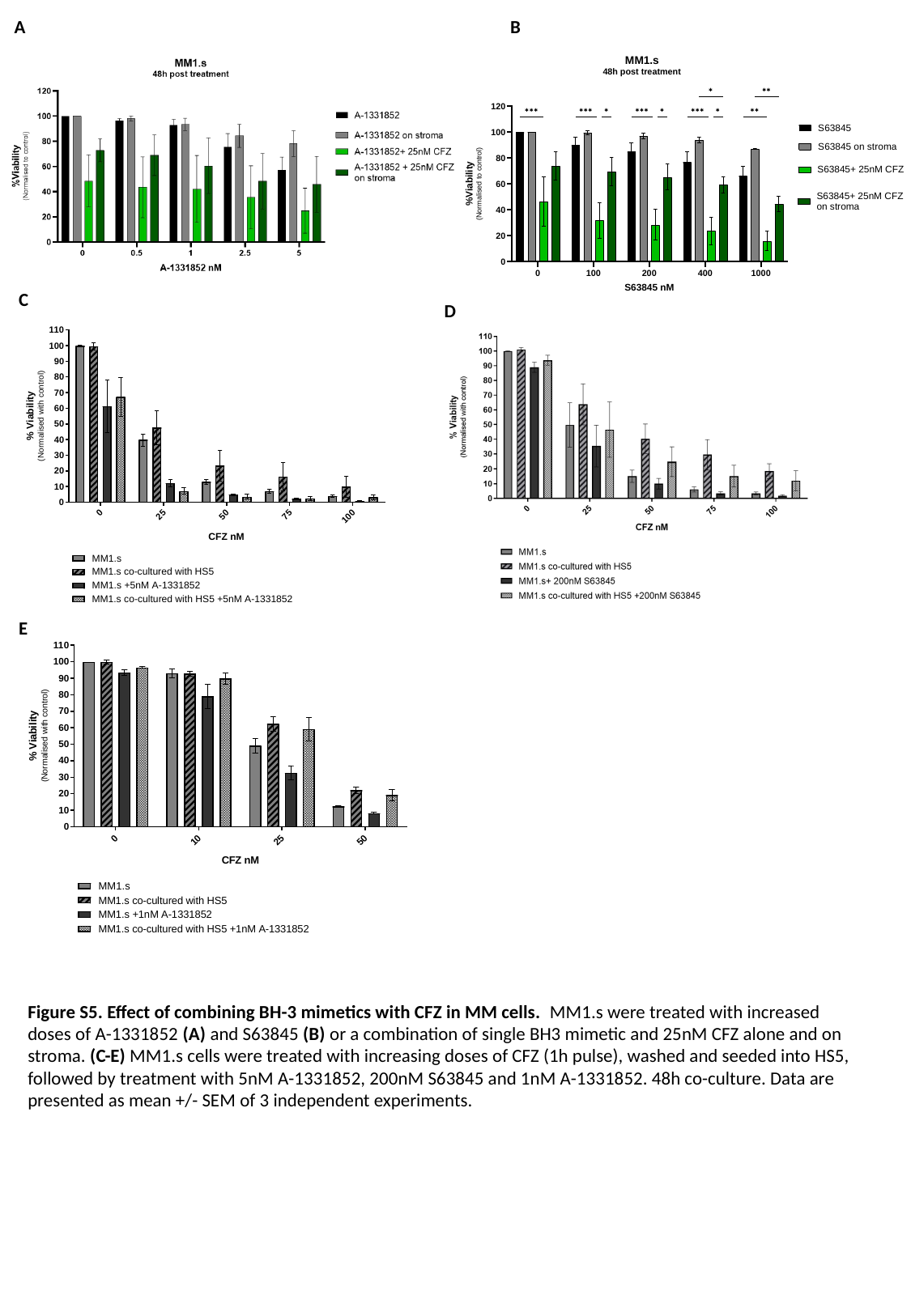

A
B
C
D
E
Figure S5. Effect of combining BH-3 mimetics with CFZ in MM cells. MM1.s were treated with increased doses of A-1331852 (A) and S63845 (B) or a combination of single BH3 mimetic and 25nM CFZ alone and on stroma. (C-E) MM1.s cells were treated with increasing doses of CFZ (1h pulse), washed and seeded into HS5, followed by treatment with 5nM A-1331852, 200nM S63845 and 1nM A-1331852. 48h co-culture. Data are presented as mean +/- SEM of 3 independent experiments.

## Slide 6
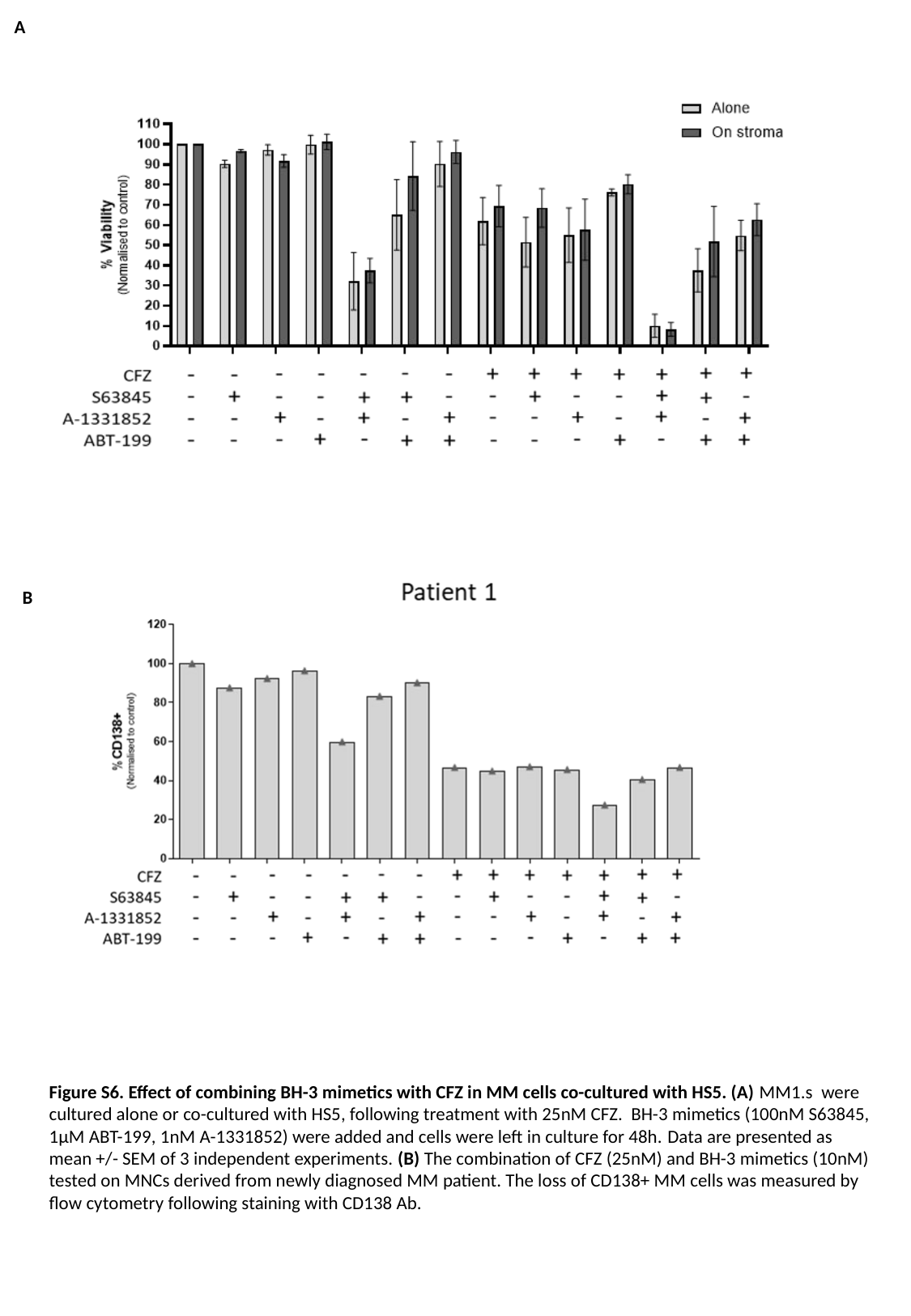

A
B
Figure S6. Effect of combining BH-3 mimetics with CFZ in MM cells co-cultured with HS5. (A) MM1.s were cultured alone or co-cultured with HS5, following treatment with 25nM CFZ. BH-3 mimetics (100nM S63845, 1µM ABT-199, 1nM A-1331852) were added and cells were left in culture for 48h. Data are presented as mean +/- SEM of 3 independent experiments. (B) The combination of CFZ (25nM) and BH-3 mimetics (10nM) tested on MNCs derived from newly diagnosed MM patient. The loss of CD138+ MM cells was measured by flow cytometry following staining with CD138 Ab.

## Slide 7
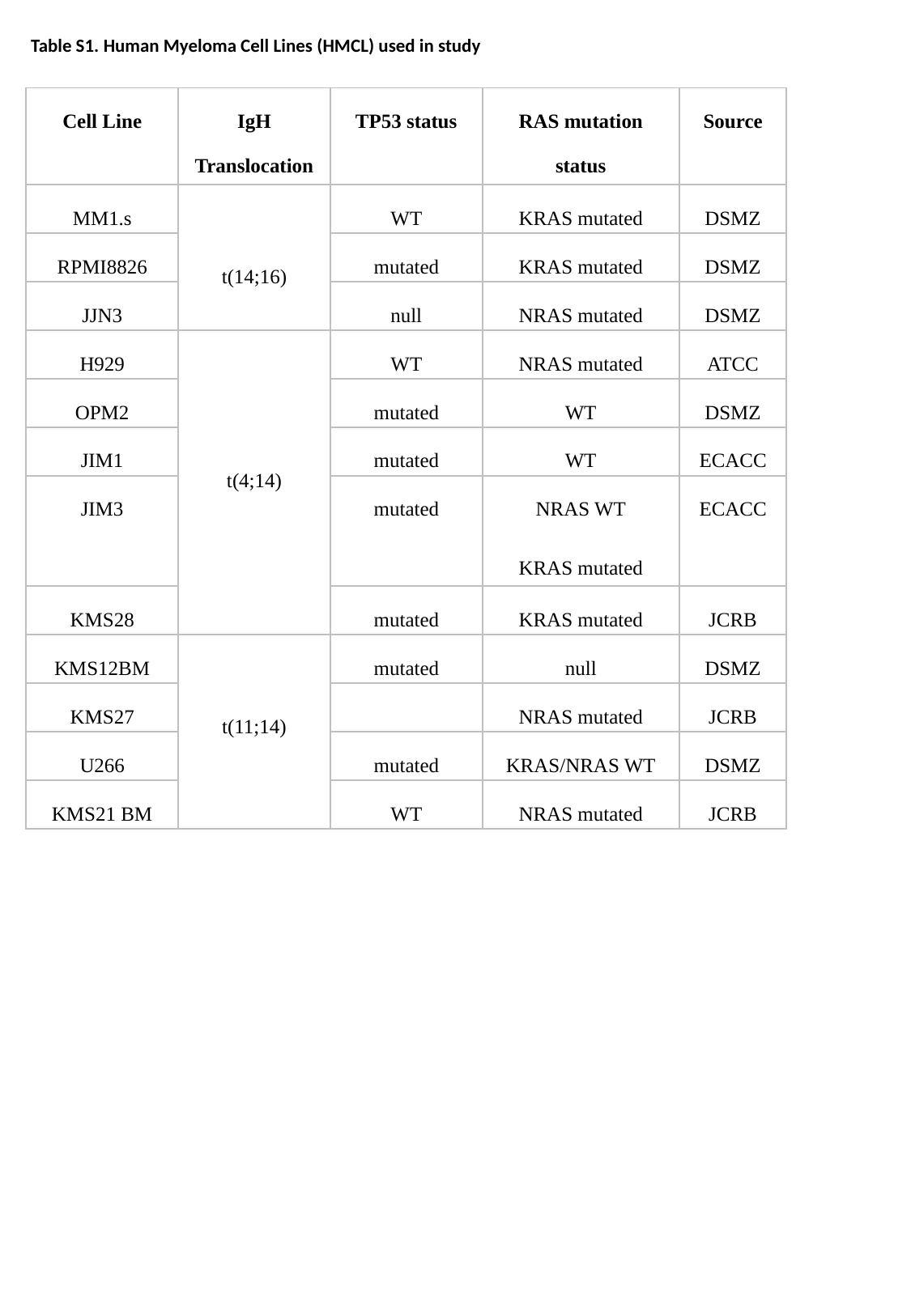

Table S1. Human Myeloma Cell Lines (HMCL) used in study
| Cell Line | IgH Translocation | TP53 status | RAS mutation status | Source |
| --- | --- | --- | --- | --- |
| MM1.s | t(14;16) | WT | KRAS mutated | DSMZ |
| RPMI8826 | | mutated | KRAS mutated | DSMZ |
| JJN3 | | null | NRAS mutated | DSMZ |
| H929 | t(4;14) | WT | NRAS mutated | ATCC |
| OPM2 | | mutated | WT | DSMZ |
| JIM1 | | mutated | WT | ECACC |
| JIM3 | | mutated | NRAS WT KRAS mutated | ECACC |
| KMS28 | | mutated | KRAS mutated | JCRB |
| KMS12BM | t(11;14) | mutated | null | DSMZ |
| KMS27 | | | NRAS mutated | JCRB |
| U266 | | mutated | KRAS/NRAS WT | DSMZ |
| KMS21 BM | | WT | NRAS mutated | JCRB |

## Slide 8
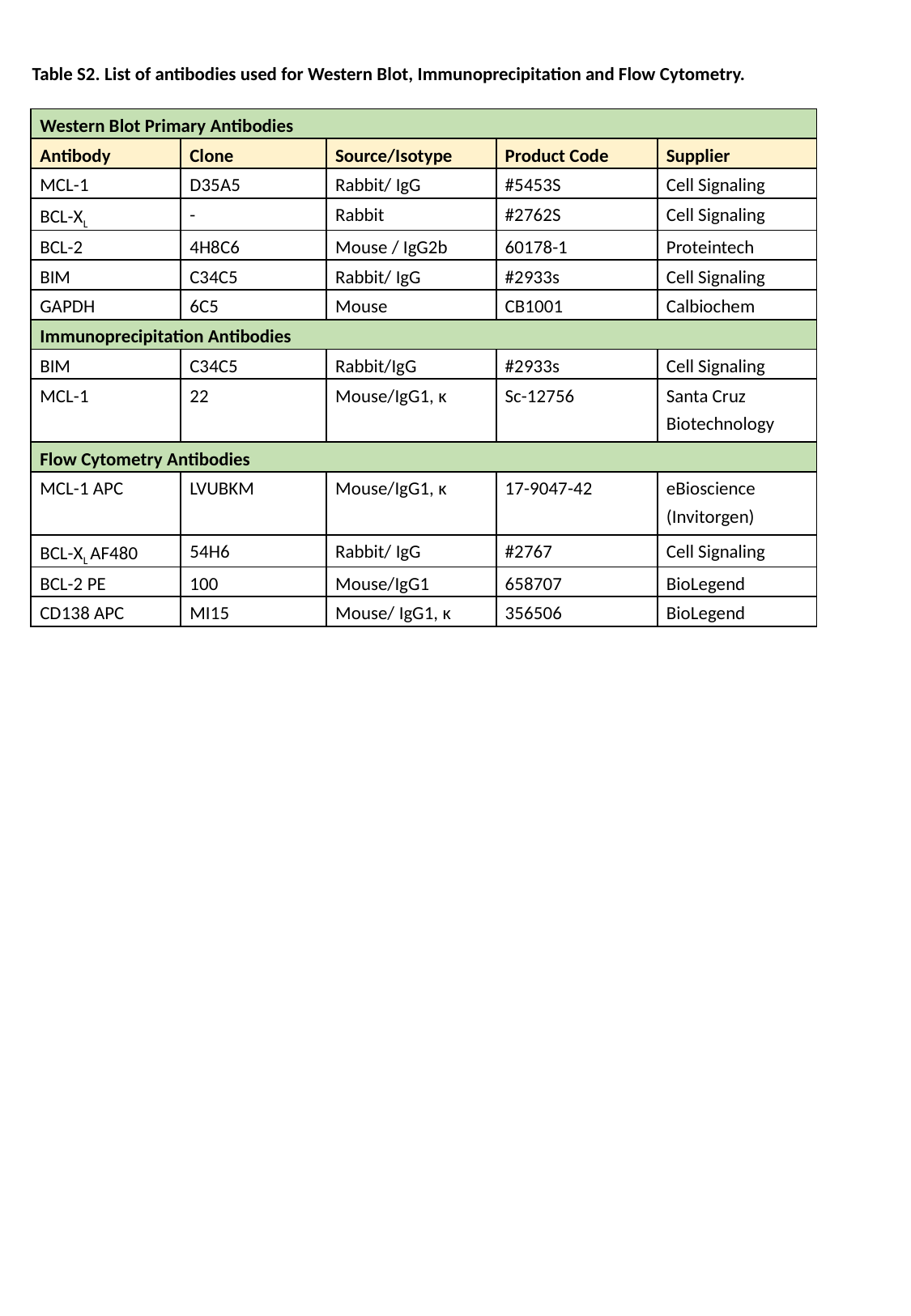

Table S2. List of antibodies used for Western Blot, Immunoprecipitation and Flow Cytometry.
| Western Blot Primary Antibodies | | | | |
| --- | --- | --- | --- | --- |
| Antibody | Clone | Source/Isotype | Product Code | Supplier |
| MCL-1 | D35A5 | Rabbit/ IgG | #5453S | Cell Signaling |
| BCL-XL | - | Rabbit | #2762S | Cell Signaling |
| BCL-2 | 4H8C6 | Mouse / IgG2b | 60178-1 | Proteintech |
| BIM | C34C5 | Rabbit/ IgG | #2933s | Cell Signaling |
| GAPDH | 6C5 | Mouse | CB1001 | Calbiochem |
| Immunoprecipitation Antibodies | | | | |
| BIM | C34C5 | Rabbit/IgG | #2933s | Cell Signaling |
| MCL-1 | 22 | Mouse/IgG1, ĸ | Sc-12756 | Santa Cruz Biotechnology |
| Flow Cytometry Antibodies | | | | |
| MCL-1 APC | LVUBKM | Mouse/IgG1, ĸ | 17-9047-42 | eBioscience (Invitorgen) |
| BCL-XL AF480 | 54H6 | Rabbit/ IgG | #2767 | Cell Signaling |
| BCL-2 PE | 100 | Mouse/IgG1 | 658707 | BioLegend |
| CD138 APC | MI15 | Mouse/ IgG1, ĸ | 356506 | BioLegend |

## Slide 9
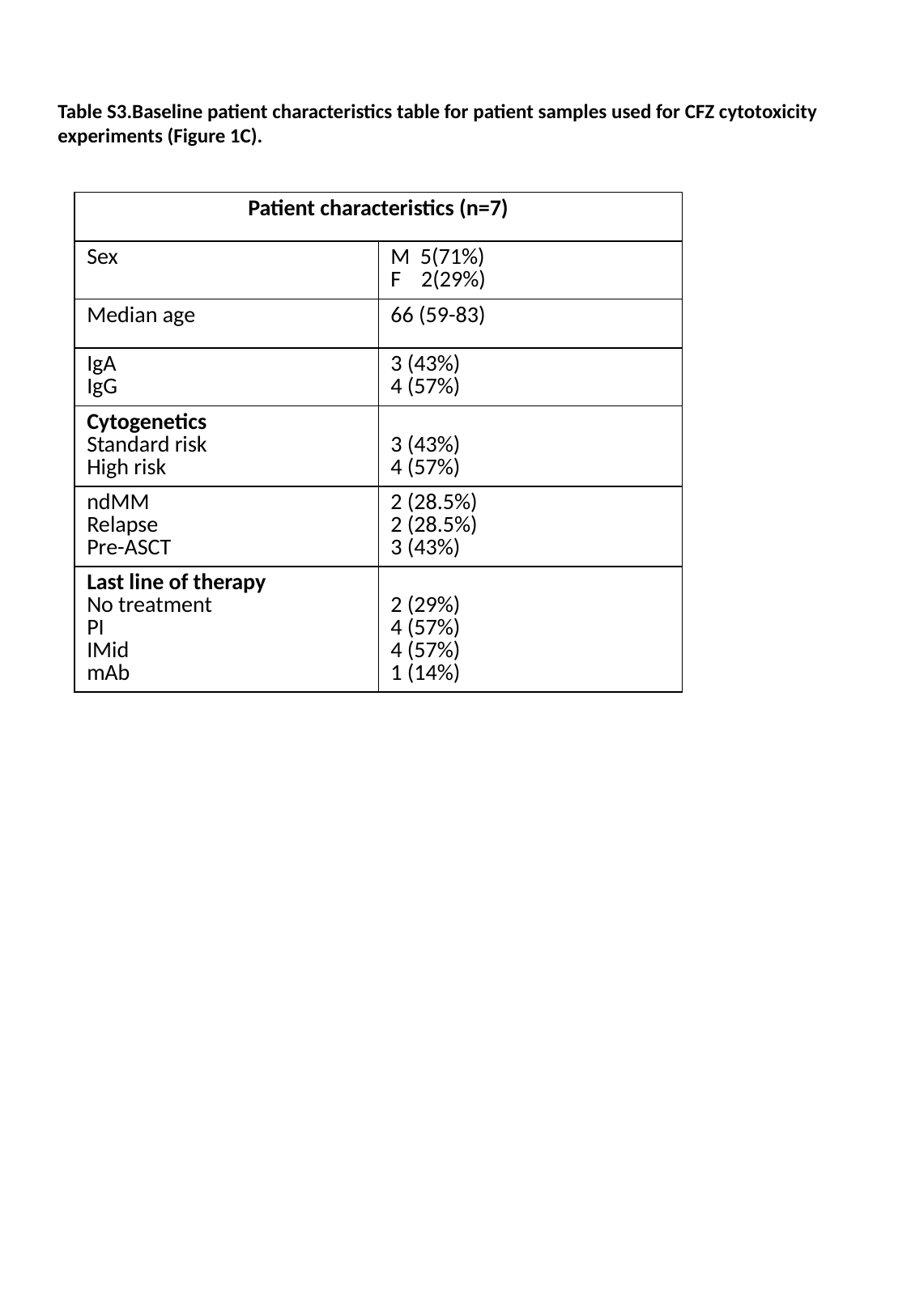

Table S3.Baseline patient characteristics table for patient samples used for CFZ cytotoxicity experiments (Figure 1C).
| Patient characteristics (n=7) | |
| --- | --- |
| Sex | M 5(71%) F 2(29%) |
| Median age | 66 (59-83) |
| IgA IgG | 3 (43%) 4 (57%) |
| Cytogenetics Standard risk High risk | 3 (43%) 4 (57%) |
| ndMM Relapse Pre-ASCT | 2 (28.5%) 2 (28.5%) 3 (43%) |
| Last line of therapy No treatment PI IMid mAb | 2 (29%) 4 (57%) 4 (57%) 1 (14%) |

## Slide 10
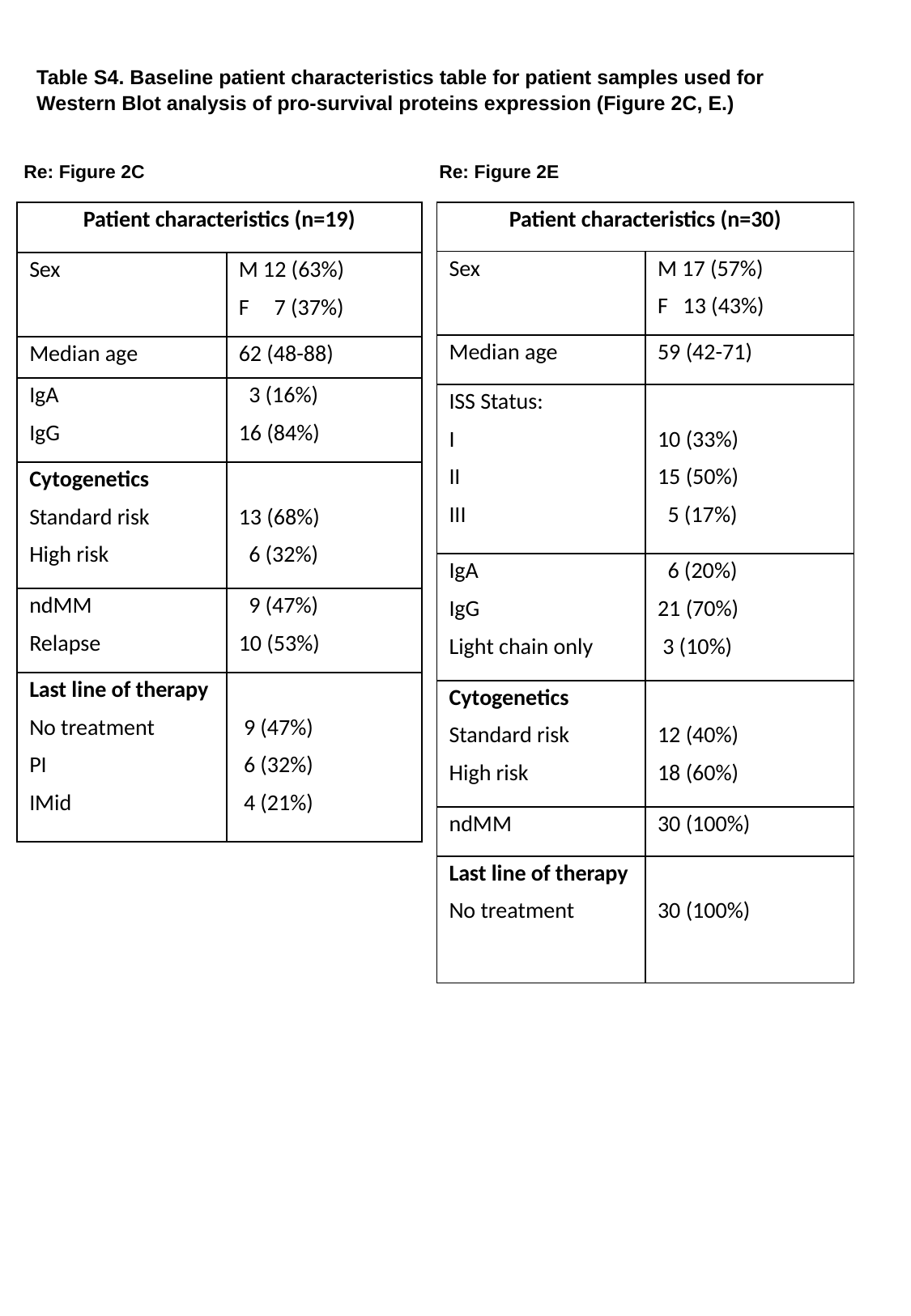

Table S4. Baseline patient characteristics table for patient samples used for Western Blot analysis of pro-survival proteins expression (Figure 2C, E.)
Re: Figure 2C
Re: Figure 2E
| Patient characteristics (n=19) | |
| --- | --- |
| Sex | M 12 (63%) F 7 (37%) |
| Median age | 62 (48-88) |
| IgA IgG | 3 (16%) 16 (84%) |
| Cytogenetics Standard risk High risk | 13 (68%) 6 (32%) |
| ndMM Relapse | 9 (47%) 10 (53%) |
| Last line of therapy No treatment PI IMid | 9 (47%) 6 (32%) 4 (21%) |
| Patient characteristics (n=30) | |
| --- | --- |
| Sex | M 17 (57%) F 13 (43%) |
| Median age | 59 (42-71) |
| ISS Status: I II III | 10 (33%) 15 (50%) 5 (17%) |
| IgA IgG Light chain only | 6 (20%) 21 (70%) 3 (10%) |
| Cytogenetics Standard risk High risk | 12 (40%) 18 (60%) |
| ndMM | 30 (100%) |
| Last line of therapy No treatment | 30 (100%) |

## Slide 11
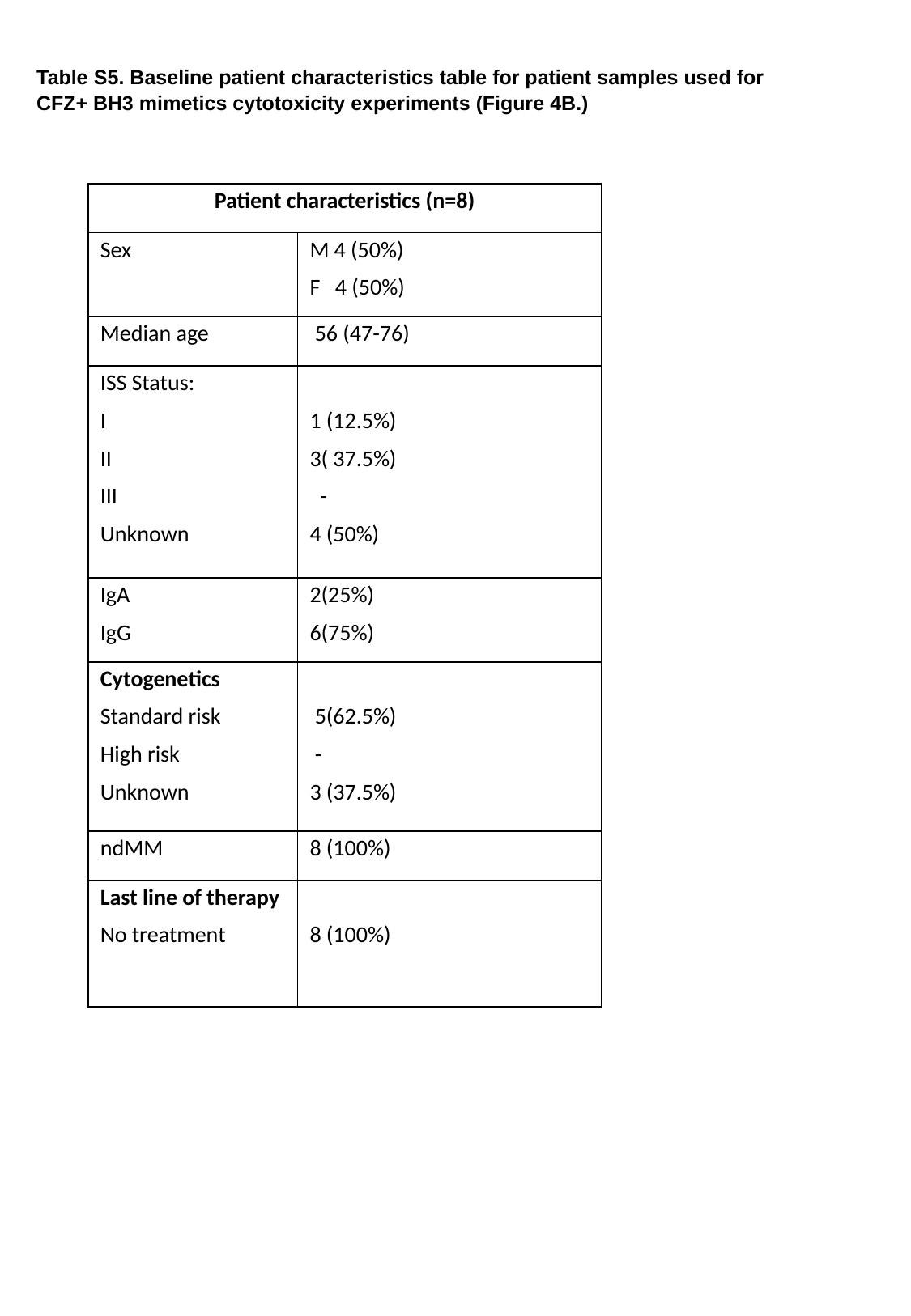

Table S5. Baseline patient characteristics table for patient samples used for CFZ+ BH3 mimetics cytotoxicity experiments (Figure 4B.)
| Patient characteristics (n=8) | |
| --- | --- |
| Sex | M 4 (50%) F 4 (50%) |
| Median age | 56 (47-76) |
| ISS Status: I II III Unknown | 1 (12.5%) 3( 37.5%) - 4 (50%) |
| IgA IgG | 2(25%) 6(75%) |
| Cytogenetics Standard risk High risk Unknown | 5(62.5%) - 3 (37.5%) |
| ndMM | 8 (100%) |
| Last line of therapy No treatment | 8 (100%) |
